# The PICASO cloud platform for improved holistic care in rheumatoid arthritis treatment—experiences of patients and clinicians

**DOI:** 10.1186/s13075-021-02526-7

**Published:** 2021-05-27

**Authors:** Jutta G. Richter, Gamal Chehab, Catarina Schwartz, Elisabeth Ricken, Monika Tomczak, Hasan Acar, Henrike Gappa, Carlos A. Velasco, Peter Rosengren, Armanas Povilionis, Matthias Schneider, Jesper Thestrup

**Affiliations:** 1 Department Rheumatology & Hiller-Research Unit Rheumatology, Heinrich-Heine-University Düsseldorf, Medical Faculty, Moorenstr. 5, 40225 Duesseldorf, Germany; 2 Fraunhofer Institute for Applied Information Technology FIT, St. Augustin, Germany; 3 CNet Svenska AB, Stockholm, Sweden; 4 Xigme OÜ, Tallinn, Estonia; 5 In-JeT ApS, Birkerød, Denmark

**Keywords:** ICT platform, eHealth, Cloud, Rheumatoid arthritis, Usability engineering, User experience design

## Abstract

**Background:**

Multimorbidity raises the number of essential information needed for delivery of high-quality care in patients with chronic diseases like rheumatoid arthritis (RA). We evaluated an innovative ICT platform for integrated care which orchestrates data from various health care providers to optimize care management processes.

**Methods:**

The Horizon2020-funded research project PICASO (picaso-project.eu) established an ICT platform that offers integration of care services across providers and supports patients’ management along the continuum of care, leaving the data with the owner. Strict conformity with ethical and legal legislations was augmented with a usability-driven engineering process, user requirements gathering from relevant stakeholders, and expert walkthroughs guided developments. Developments based on the HL7/FHIR standard granting interoperability. Platform’s applicability in clinical routine was an essential aim. Thus, we evaluated the platform according to an evaluation framework in an observational 6-month proof-of-concept study with RA patients affected by cardiovascular comorbidities using questionnaires, interviews, and platform data.

**Results:**

Thirty RA patients (80% female) participated, mean age 59 years, disease duration 13 years, average number of comorbidities 2.9. Home monitoring data demonstrated high platform adherence. Evaluations yielded predominantly positive feedback: The innovative dashboard-like design offering time-efficient data visualization, comprehension, and personalization was well accepted, i.e., patients rated the platform “overall” as 2.3 (1.1) (mean (SD), Likert scales 1–6) and clinicians recommended further platform use for 93% of their patients. They managed 86% of patients’ visits using the clinician dashboard. Dashboards were valued for a broader view of health status and patient-physician interactions. Platform use contributed to improved disease and comorbidity management (i.e., in 70% physicians reported usefulness to assess patients’ diseases and in 33% potential influence on treatment decisions; risk manager was used in 59%) and empowered patients (i.e., 48% set themselves new health-related goals, 92% stated easier patient-physician communications).

**Conclusion:**

Comprehensive aggregation of clinical data from distributed sources in a modern, GDPR-compliant cloud platform can improve physicians’ and patients’ knowledge of the disease status and comorbidities as well as patients’ management. It empowers patients to monitor and positively contribute to their disease management. Effects on patients’ outcome, behavior, and changes in the health care systems should be explored by implementing ICT-based platforms enriched by upcoming Artificial Intelligence features where possible.

**Trial registration:**

DRKS—German Clinical Trials Register, DRKS00013637, prospectively registered. 17 January 2018.

## Introduction

Multimorbidity has an increasing impact on health care systems especially in aging and developed countries and will further evolve [1, 2]. Due to the complexity and the severity of the diseases and their combinations, these patients require substantially more resources and still have a markedly lower quality of life than most patients with just one chronic disease [3]. This is particularly important for patients with inflammatory rheumatic diseases who experience a high risk of significant comorbidities during lifetime potentially ending up in polypharmacy. Thus, when making treatment adaptations consequent, management of comorbidities is as decisive as good control of the inflammatory rheumatic disease [4–6].

However, data of a patient’s different diseases and their treatments are usually collected from various health-service providers and are not readily available to each health care provider in the treatment chain for treatment decisions in time. Often, information (e.g., on health status assessment, pathway decisions) relies on patients` memory or written medical reports which do not cover all relevant aspects and which may not be fully available.

The orchestration of available information for a patient in the continuum of care—consisting of hospitals, outpatient departments, practices, non-physician health-service providers, home monitoring—into a comprehensive view would enable a more efficient and effective use of available data. This process should be personalized and patient-centered, and it forms the blueprint for the development of new technology-enabled care models for the management of multimorbidity.

Within the Horizon2020-funded project PICASO, we developed an information and communication (ICT) platform that offers a solution for these complex issues. The development was driven by user requirements and modern IT development–standards in eHealth (https://www.picaso-project.eu). Considered key aspects were the platforms’ usability in patients’ environments and in daily clinical routine. The platform supports collaborative sharing of care plans and data across care providers based on dynamic and personalized orchestration of services. An evaluation framework adapted to the technical developments was established to evaluate the platform in a 6-month proof-of-concept study in clinical routine care with rheumatoid arthritis (RA) patients and their caring physicians. We report results with respect to acceptance, clinical relevance, usability, user experience, and user satisfaction.

## Material and methods

### PICASO platform

**Fig. 1:**
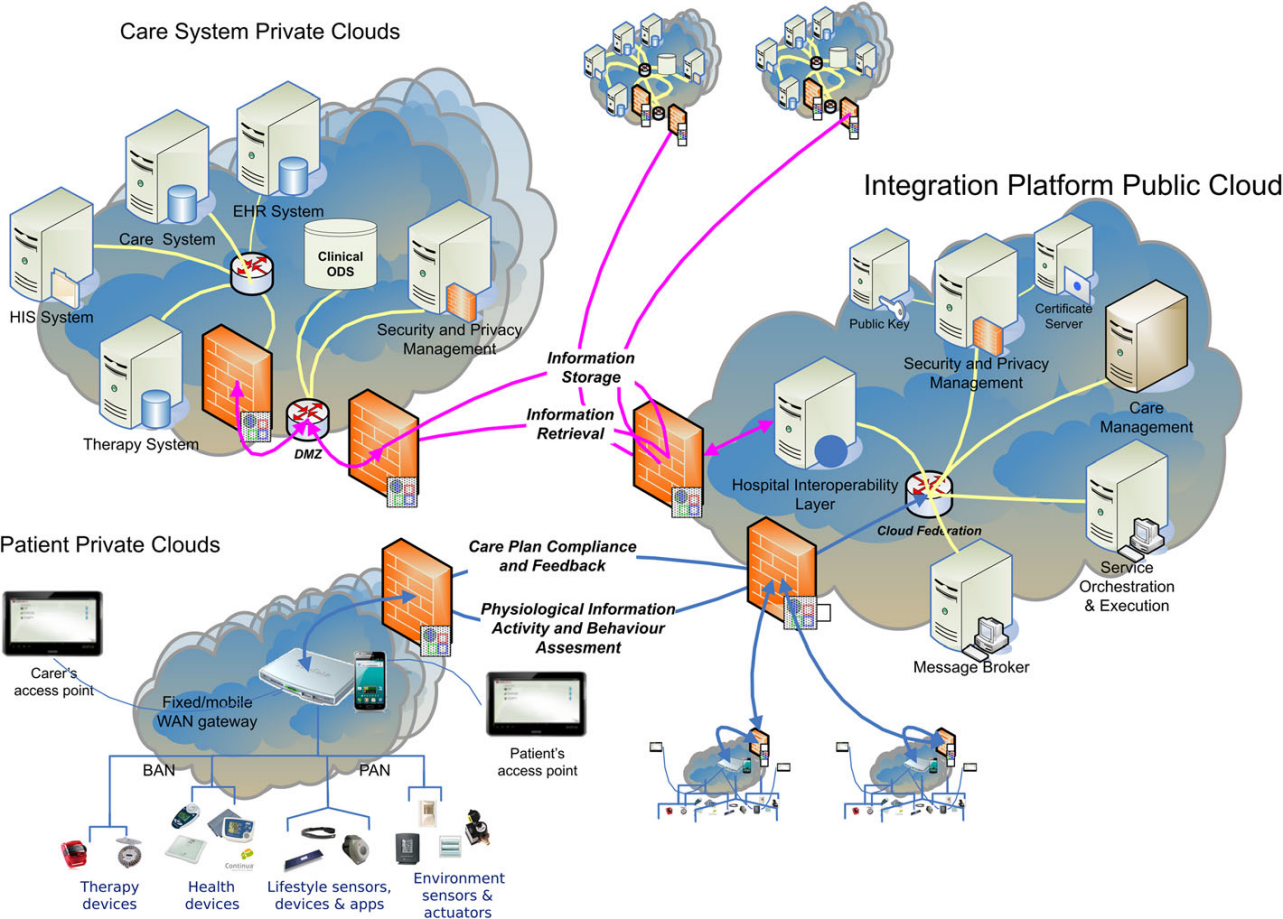
PICASO platform architecture

The PICASO platform was developed within a Horizon2020-funded project between 2016 and 2019. Details of the underlying platform architecture, which especially addressed identity management, access control, and privacy aspects ensuring that the data remains with the owner, have been published elsewhere [7] and are depicted in Fig. 1. Conformity with the European General Data Protection Regulation (GDPR) as well as national regulations was precisely adhered to [8]. All ICT interface developments were based on the new HL7/FHIR (“Fast Healthcare Interoperability Resources”) standard in order to enable data exchange with other software systems in the health care sector.

User requirements (*n*=119) were identified in focus groups comprising various stakeholders (e.g., patients, physicians, study nurse, health care insurance) and patient interviews. The requirements included a broad spectrum with functional and non-functional requirements (e.g., “look and feel,” performance, security, ethical, societal). Exemplary requirements were as follows: (1) Patients and physicians should be presented an integrated view on patients’ self-reported data and home monitoring measurements (also in retrospective). (2) Provision of an advanced risk assessment. (3) Easy and time-efficient use. The collected user requirements provided the basis for PICASO’s human-centered design approach and the development of the evaluation framework [9]. “Group-based expert walkthroughs” on usability and utility issues resulted in a constantly updated requirements’ document serving as a reference for application development [10].

**Fig. 2:**
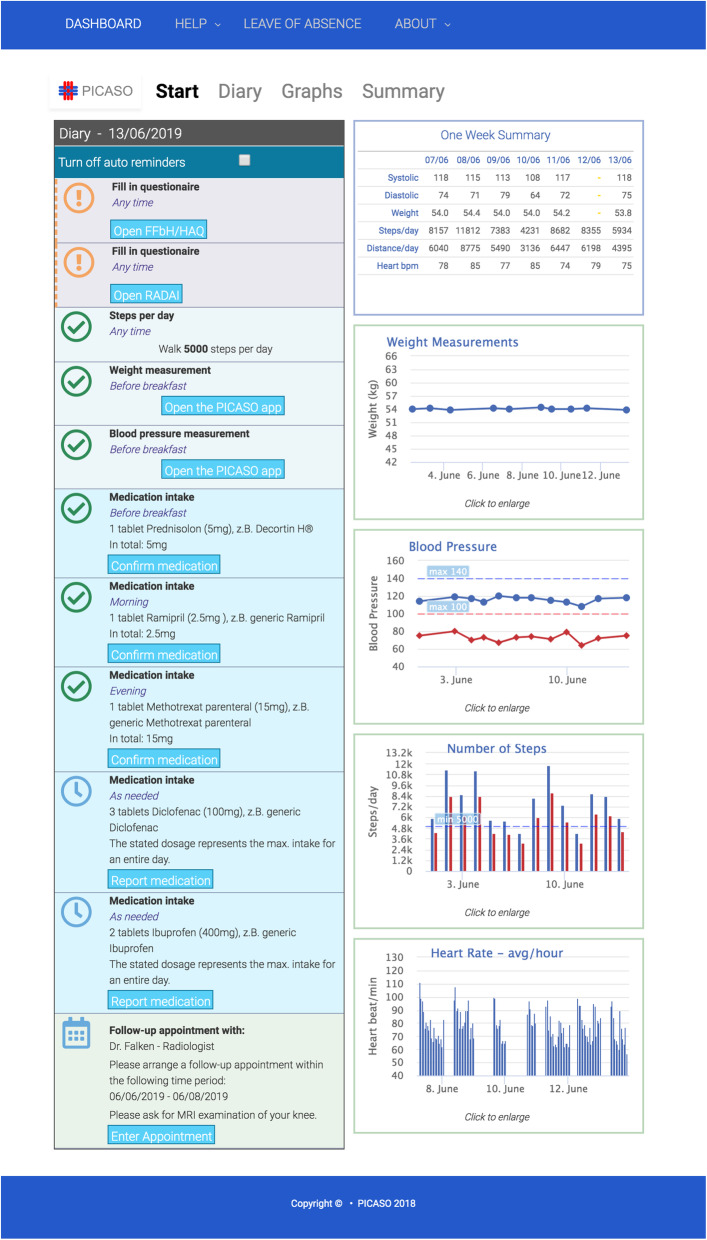
Start page patient dashboard

**Fig. 3:**
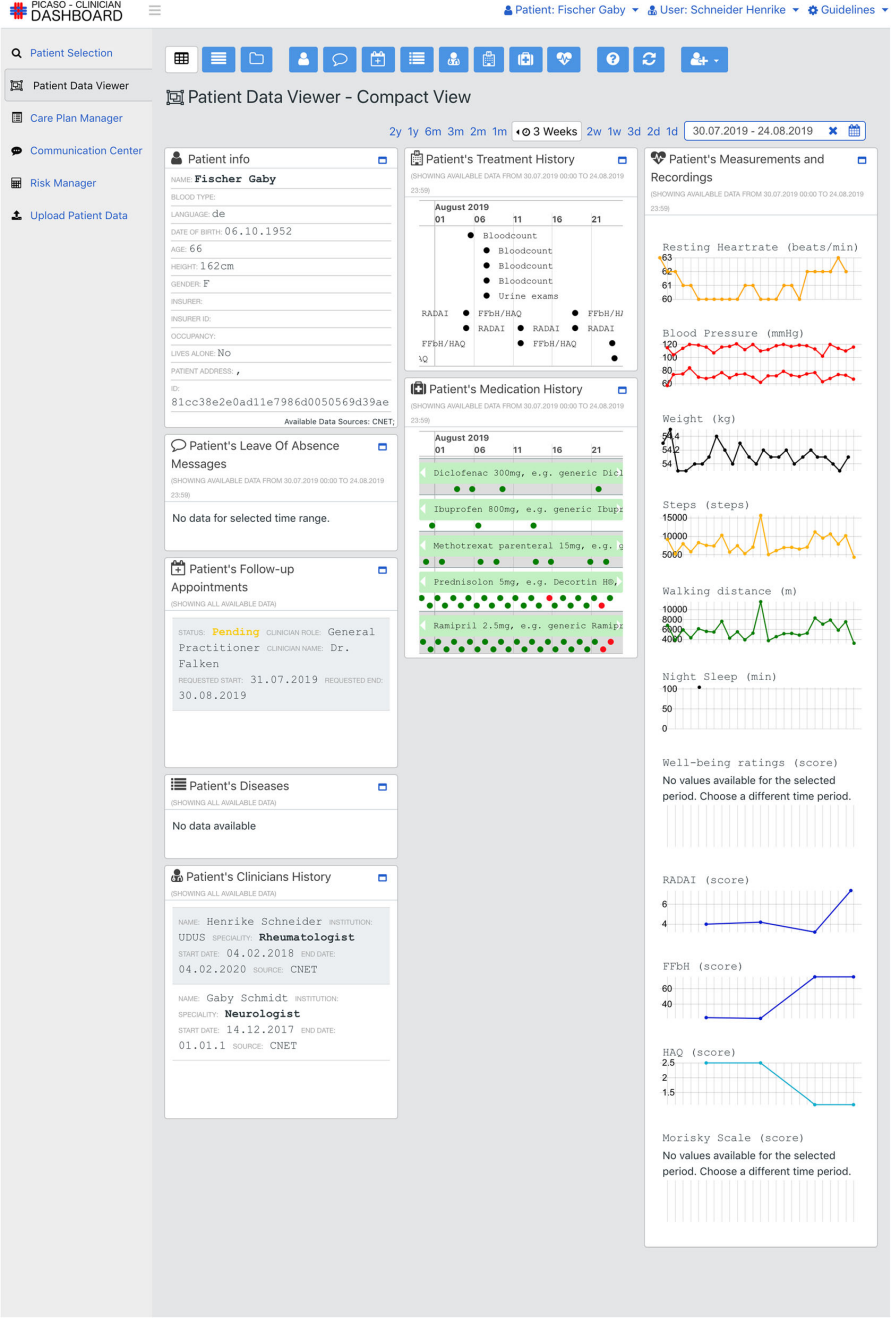
Clinician dashboard depicting patients’ data

The development resulted in a patient (PD) and clinician dashboard (CD). Both offered a configurable overview of the patients' daily care plan and progress of health status using graphs (see Figs. 2 and 3). PD was designed as presenting patients’ daily tasks as well as results of health measurements and self-recordings on one page (see Fig. 2). The CD visualization included home monitoring data as well as information provided from other stakeholders at a glance and was easily customizable to clinicians’ individual needs. It allowed them to create and change care plans, use the communication center, and access a risk manager for cardiovascular disease risk assessment (e.g., Systematic COronary Risk Evaluation, SCORE) [5, 11].

### Study processes

Thirty patients were recruited consecutively from our outpatients’ clinics. Inclusion criteria were age above 18 years of age, diagnosis of RA (ICD-10-Code M05.* or M06.*) with at least one known cardiovascular comorbidity, and good German skills. Patients’ signed informed consents were obtained. Furthermore, nine correspondingly caring physicians (seven rheumatologists and two general practitioners (GP)) were included.

For remote health monitoring, patients received devices to self-track their blood pressure and weight (medical products: A&D UA-651 BLE and A&D UC-352 BLE 200kg) as well as daily activities (fitbit charge 2™). The devices transmitted data via Bluetooth to a commercially available Android™ tablet with an integrated SIM LTE card that was also handed out from the study team. Home monitoring data were transferred through the PICASO Integration Platform. In addition, patients used the tablet to access their individual PICASO PD for data entry and monitoring their health status.

Validated patient-reported outcome measures (PROMs) were used as electronic forms in the PD: Functional capacity was measured by the Hannover Functional Ability Questionnaire (FFbH), and its values were derived to Health Assessment Questionnaire (HAQ) values [12] and are reported as this below. Self-reported disease activity was assessed via the RA Disease Activity Index (RADAI) [13]. Patients’ clinical and sociodemographic data were recorded according to the standardized processes at our clinic (e.g., Disease Activity Score (DAS) 28 CRP, medication) at baseline and follow-up visits (after 3 and 6 months).

Patients performed a standardized, 1-h user training on the PD at baseline. All received a paper-based user manual. Telephone and email contact to the local PICASO hotline was available during business hours. All patients agreed to perform blood pressure measurements, weighing, fitbit charge 2™ use, and ePROMs documentation on a regular basis. At baseline, the treating physician and the patient agreed upon the frequency which became part of their individual care plan. Reporting the medication intake was part of the tasks depicted daily in the PD. Reporting as specified was appreciated but still voluntary and not regularly controlled until the next outpatient visit.

Physicians were trained for the PICASO platform by the study investigators. They reviewed available data in the CD during follow-up visits and could use its functionalities.

### Evaluation concept

Patients and physicians evaluated the platform after 3 and 6 months according to the evaluation framework [9] assessing the acceptability, usability, user satisfaction, and clinical relevance of the platform through (self-)developed paper-based questionnaires. Patients’ prior technical knowledge and their expectations in relation to their use of the PICASO platform were evaluated at baseline with an established questionnaire [14]. Gathering feedback on potential usability issues was an important part of the formative evaluation and of the user-centric approach applied in the project. Additional semi-structured interviews were conducted with 20 patients collecting information about well-established usability principles and other key factors determining the ease of use of a system [15].

When new applications are launched and comparisons to previous versions are impossible, a relevant question is whether the user experience (UX) is sufficient to meet user expectations [16]. UX is a relevant aspect of the success of a product [17]. Thus, the “User Experience Questionnaire” (UEQ), an established, fast, and reliable 26-item questionnaire, measured UX. The questionnaire items consist of pairs of terms with opposite meanings (e.g., efficient-inefficient) on 7-point Likert scales, transformed to −3 (fully agree with a negative term) respectively to +3 (fully agree with a positive term) values. The items are then grouped into six scales (attractiveness, perspicuity, efficiency, dependability, stimulation, and novelty). Scale values between −0.8 and 0.8 represent a neutral evaluation of the corresponding category, values > 0.8 a positive evaluation, and values < −0.8 a negative evaluation; observation of values above +2 or below −2 are extremely unlikely [18]. Thus, the UEQ includes relevant UX quality aspects such as usability (efficiency, perspicuity, dependability) and further user experience aspects (originality, stimulation) respectively pragmatic and hedonic quality aspects [https://www.ueq-online.org/] [17, 18].

### Ethical and administrative issues

Positive approval of the ethics committee of the Medical Faculty of the Heinrich Heine University Duesseldorf and of the local data security officers was obtained (local ethics study number 6139R). The study was registered to the German Clinical Trials Register (Identifier DRKS00013637).

### Statistical analyses

As this was a proof-of-concept study, the sample size was determined to consider limitations due to available EU funding, yet allow for statistical analysis. Two-pass verification was performed to reduce data entry errors for the digitization of the evaluation questionnaires. Data were then imported into IBM SPSS Statistics 25 (IBM Corp., Armonk, NY, USA) for statistical analyses. Predominantly descriptive statistics were executed. Values are expressed as valid percentages for discrete variables, or as mean (standard deviation (SD)) or median for continuous variables. UEQ measures were calculated using the Data Analysis Tool provided by the UEQ Team (see https://www.ueq-online.org/). As UEQ data were measured twice (after 3 and 6 months), resulting in pairs of observations, paired *T*-tests were applied for more detailed UEQ analyses. All statistical tests were performed two-tailed; *p*-values less than 0.05 were considered significant.

## Results

Eighty RA patients were screened to recruit 30 participating patients. Non-participants (*n*=50) were 65.0 (10.5) years old, 64% (*n*=32/50) female, had a disease duration of 11.1 (8.3) years (mean (SD)), DAS28 CRP was 2.5 (0.8) (mean (SD)), number of comorbidities 3.6 (2.4) (mean (SD)), and mean (SD) HAQ score was 1.13 (0.66). Participating patients’ clinical, sociodemographic, and IT knowledge data are summarized in Tables 1 and 2. Nine physicians participated in the evaluation at baseline. Seven of them were rheumatologists (78%, *n*=7/9) and two GPs (22%, *n*=2/9), 33% (*n*=3/9) female. Five physicians were < 50 years old at baseline, three 50 to 59 years old, and one >60 years old. Physicians had been active for 18 years in their respective specialties (median).

**Table 1:** Patients’ clinical, sociodemographic, and IT knowledge data as well as physician-related data

	Patients
*Age in years (mean (SD))*	58.6 (10.8)
*% Female gender (n)*	80 (24/30)
*Disease duration in years (mean (SD))*	12.6 (8.5)
*No. of comorbidities (mean (SD))*	2.9 (1.6)
*HAQ* (*mean (SD))*	0.97 (0.65)
*DAS28(CRP) (mean (SD))*	2.7 (1.0)
*Medication*	
*% oral glucocorticoids (n)*	40 (12/30)
*% csDMARD alone or in combination with bDMARD or tsDMARD (n)*	73 (22/30)
*% bDMARD OR tsDMARD alone (n)*	13 (4/30)
*% NSAIDs/Coxibs (n)*	33 (10/30)
*Comorbidities*	
*% Arterial hypertension (n)*	53 (16/30)
*% Hypercholesterineamia (n)*	20 (6/30)
*% Diabetes (n)*	20 (6/30)
*% Coronary heart disease (n)*	10 (3/30)
*% Obesity (n)*	13 (4/30)
*Education/working situation*	
*% University degree (n)*	37 (11/30)

**Table 2:** Patients’ and physicians’ IT knowledge and experience data

	Patients	Physicians
*IT knowledge*		
*% Smartphone experience (n)*	93 (28/30)	100 (9/9)
*Use since years (mean (SD))*	7.9 (5.8)	8.2 (4.2)
*% Tablet experience (n)*	70 (21/30)	67 (6/9)
*Use since years (mean (SD))*	6.0 (3.4)	8.5(4.0)
*% Computer use (n)*	73 (22/30)	100 (9/9)
*% Notebook use (n)*	60 (18/30)	100 (9/9)
*Internet experiences*		
*% Internet use (n)*	90 (27/30)	100 (9/9)
*Use since years (mean (SD))*	12.6 (5.5)	18.1 (6.9)
*Use/day in hours (median)*	1.9 (2.3)	4.4 (4.4)
*% Internet use on smartphone (n)*	83 (25/30)	89 (8/9)
*% Internet use on tablet (n)*	57 (17/30)	56 (5/9)
*% Internet use on personal computer respectively notebook (n)*	47 (14/30)	89 (8/9)
*Confidence in the Internet (mean (SD)), Likert 1 (very high) to 6 (very low)*	3.3 (1.4)	2.6 (0.9)
*Reliability of information retrieved from the Internet (mean (SD)), Likert 1 (very high) to 6 (very low)*	3.2 (1.1)	3.1 (0.6)
*% Use of WiFis at home (n)*	93 (28/30)	100 (9/9)
*% Use of public WiFis (n)*	20 (6/30)	67 (6/9)
*% Social media use (n)*	30 (9/30)	11 (1/9)
*% Pre-existing experience with clouds (n)*	67 (20/30)	44 (4/9)
*Security of data in clouds (mean (SD)), Likert 1 (very high) to 6 (very low)*	3.7 (1.2)	3.9 (0.9)

*SD* standard deviation

### Platform use

Over the 6 months, adherence to the platform was high as there was only one drop out (early after 4 days). Overall, patients’ project participation and thus potential use of the platform was 180.1 (14.6) days (mean (SD)) respectively 25.7 (2.1) weeks (mean (SD)). Table 3 lists details of the home monitoring including ePROMs filled via the PD over the complete study period. The high number of measured steps per patient over the study time (median 1,012,314) needs emphasis.

**Table 3:** Patient-related data retrieved from the PICASO ODS at UDUS

Parameter recordings over the project period per patient	Median	Maximum	Average	% of expected recordings over 6 months
Number of filled eFFbH/HAQ Questionnaires	21	83	24	92
Number of filled eRADAI Questionnaires	22	84	29	112
Days of daily steps count (source fitbit charge 2™)	150	268	152	84
Heart rate resting (source fitbit charge 2™)	151	266	143	79
Weight	150	256	149	82
Systolic/diastolic blood pressure measurements (source blood pressure device)	307	478	276	51
Pulse rate (source blood pressure device)	307	478	276	51
**Activity**				
Total number of steps during trial per patient (source fitbit charge 2™)	1,012,314	3,104,342	1,203,094	132
**Reporting frequency**				
Number of daysreporting blood pressure measurements	149	186	148	81
Number of days reporting weight	146	177	142	78
Number of days reporting medication^a^	134	182	108	59

^a^Not all patients needed to report medication every day

After 6 months, 86% (*n*=25/29) of the patient’s visits were managed by the physicians using the CD. Mainly technical reasons as “the platform or data were not accessible” and only in one case “lack of time” were given for non-use. The patient data viewer was the most frequently used view (86% (*n*=25/29)). Patients’ provided blood pressure and weight values were most commonly looked at by the physicians (86% (*n*=25/29) respectively 79% (*n*=23/29)), followed by step counts and resting heart rate (each 69% (*n*=20/29)), eRADAIs (72%, *n*=21/29), eFFbH (59%, *n*=17/29), and derived HAQs (41%, *n*=12/29). The risk manager was used by the physicians in 59% (*n*=17/29) of the patients.

### Platform evaluation—patients’ view

Eighty-nine percent of the patients (*n*=24/27) were satisfied with the PD. The more detailed proxies for user satisfaction were the “overall ratings of the platform” and the “ease of use” after 3 and 6 months; they scored 2.5 (1.2) and 2.2 (1.4) (Likert scale from 1=very good to 6=very bad) respectively 2.3 (1.1) and 2.3 (1.2) (mean (SD)). Furthermore, 41% (*n*=12/29) felt better understood by their rheumatologist regarding their complaints, 97% (*n*=28/29) reported a better understanding of their complaints by their practicing physician, and 92% (*n*=22/24) reported an easier communication with the treating rheumatologist. In our semi-structured interviews, 55% (*n*=11/20) of the patients reported a better overview of their current health status by using the PD and 90% (*n*=18/20) noted that they had a good overview on their daily tasks, suggesting that majority of patients did not feel overwhelmed by the presented information. Over the complete study period, 48% (*n*=14/29) had set themselves new goals regarding their health.

At the end of the project, the majority of patients (93%, *n*=27/29) would recommend the platform to others, and about one-fifth (19%, *n*=5/27) would like to continue using the platform even if it would become subject to charge. At study end, 79% (*n*=23/29) had talked to family members and friends, of these 64% (*n*=14/22) reported positive feedback towards PICASO. Thirty-five percent (*n*=10/29) had talked to their physicians about PICASO, of these 70% (*n*=7/10) reported positive physician-based feedback.

Seventy-six percent (*n*=22/29) of the patients perceived the time expenditure for the documentation of the health data as “appropriate” and 21% (*n*=6/29) as “too high.”

### Platform evaluation—physicians’ view

At the final assessment, 75% (*n*=21/28) of the clinicians were satisfied with the CD. The usefulness of the risk manager function was rated best with 2.3 (1.0), followed by the data viewer with 2.6 (1.0) (Likert scale from 1=very high to 6=very low). The overall benefit of the additional health data for the RA treatment in these patient visits was rated 2.3 (0.8) (Likert scale from 1=very high to 6=very low). Furthermore, the overall benefit of the additional patient data for the treatment of cardiovascular comorbidities was regarded as 1.9 (0.6).

Patients’ additional health data shared and provided in the CD via the PICASO platform helped the physicians in 70% (*n*=19/27) of patients’ visits in month 6 to full and in 19% (*n*=5/27) to some extent to assess the course of patients' disease. Regarding 33% (*n*=9/27) of their patients, they stated that it might have influenced their therapeutic decision. In most patients’ visits (82%, *n*=22/27), the CD use did not cause more work than benefit.

### User experience (UEQ)—patients and physicians

**Fig. 4:**
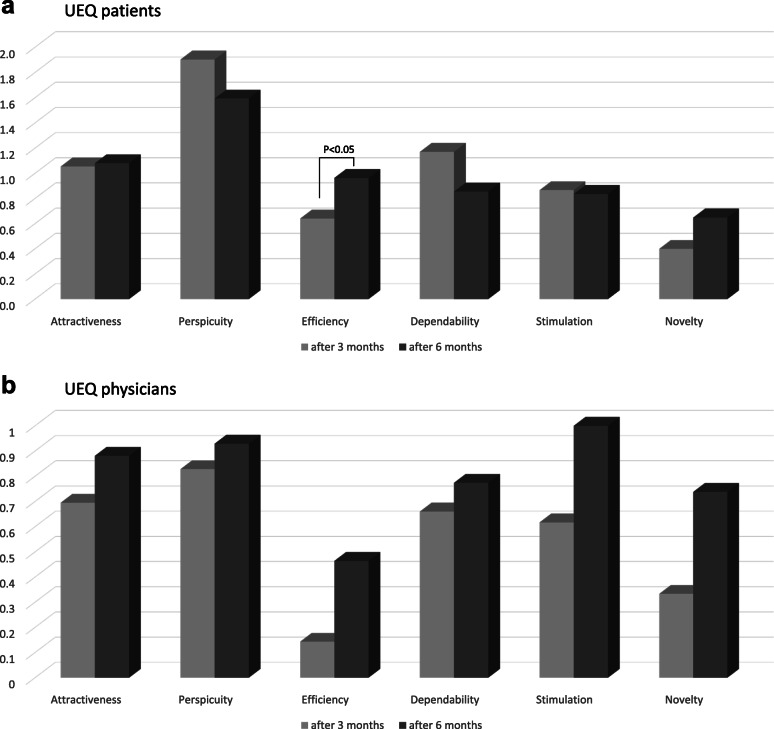
UEQ results of **a** patients’ and **b** physicians’ evaluations after 3 and 6 months. In the graphs, the abscissa shows the six evaluation categories for usability and experience aspects and the ordinate shows their evaluation. Values between −0.8 and 0.8 represent a neutral evaluation of the corresponding category, values > 0.8 a positive evaluation, and values < −0.8 a negative evaluation. **a** Patients’ evaluation of the patient dashboard. **b** Physicians’ evaluation of the clinician dashboard

UEQ scale results after 3 and 6 months are outlined in Fig. 4a (patients) and b (physicians). At each evaluation, perspicuity was rated excellent. Over time, gains in attractiveness, efficiency, and novelty were notable for patients. This increase was statistically significant for efficiency. With physicians, increases were recorded for attractiveness, perspicuity, efficiency, dependability, stimulation, and novelty. However, no statistically significant changes were notable.

### Additional feedback—patients and physicians

Five patients experienced accessibility problems when using the PD. Technical aids (e.g., a touch pen to operate the surface of the tablet) solved the issues. No problems due to RA were reported. In our semi-structured interviews, 67% (*n*=12/18) of the patients who used this functionality noted that medication confirmation was easy to do and 17% (*n*=3/18) stated that slow performance of PD hindered efficient use of this functionality. Too slow performance was reported most often as the drawback of the system, in the last evaluation questionnaire by 96% (*n*=23/24). Issues referring to data transfer from the home monitoring devices to PD were stated by (52%, *n*=14/27) and distributed uniformly on blood pressure measurements (17%, *n*=5/29), scale (28%, *n*=8/29), and t fitbit charge 2™ (21%, *n*=6/29).

In the interviews, participants were inquired about suggestions for improvement. Predominantly participants advocated for more feedback from the system about what it was doing, e.g., loading or processing information. Moreover, they requested additional vital sign measurements such as oxygen saturation or blood sugar values. The ability to store referral letters on the PD and easily share these with other medical professionals was also mentioned as well as documentation of relevant incidents like surgeries and changes in the disease status. This feature was already offered as part of the CD. Physicians raised additional points to consider, e.g., “better decision support,” “alerts for physicians when patients show values above resp. below thresholds as defined in their care plan.”

## Discussion

In recent years, a correlation between medical outcome and quality of care has been demonstrated for various chronic conditions including inflammatory rheumatic diseases [19, 20]. Multimorbidity is one of the greatest health-related challenges especially in chronic diseases typically requiring close co-operation of a multitude of specialized health care providers. It has a detrimental impact on quality of life, treatment (risks), and mortality and is associated with increased health care utilization [21–24]. Existing health care structures lead to scattered information. Bringing together these fragmented information (i.e., in a platform) is crucial to ensure quality-assured effective and efficient long-term management of multiple chronic comorbid conditions. According to our Horizon2020 project, the PICASO platform is capable of meeting these complex requirements, thus making a great and relevant contribution to a more holistic care of RA patients.

In our study, nearly all patients reported easier communication with the treating rheumatologist and a majority felt that their PD gave them a better overview of their health status. This supports research findings from Navarro-Millan et al., who reported that RA patients may be open to electronic collection and sharing of PRO data between clinical outpatient visits, if communication with health care providers is facilitated and medical feedback is given [25]. Our patients also valued the platform for their communication with others (e.g., GP, family, friends). These findings are in line with the evaluation of a dashboard that visualizes PROs in RA during outpatients’ visits, where patients emphasized that apart from understanding their disease they appreciate to share disease experiences with others [26]. Our mobile available PD could be taken to others to present and discuss the course of disease and its comorbidities.

Physicians especially valued the enhanced information (like risk assessment and home monitoring) that enabled a new comprehensive view of the patients and their comorbidities and may support treatment decisions. The data offered were used even though they are not yet officially recommended as standard of care. Patients willingly provided the data, although given tasks were clearly above the usual clinical standard [25]. This demonstrates the ability to integrate comprehensive tasks into patients’ daily lives using modern ICT solutions. Apart from improved RA outcomes, the use of the ICT platform might also improve cardiovascular outcome through the measured vital signs and the corresponding already depicted personalized upper and lower limits in the PD and the CD but also via, i.e., early warning messages that could pop-up when using the dashboards’ functionalities.

No patient reported being limited by the RA in terms of use. The active use is dependent on the quality of the platform (i.e., responsiveness, graphical user interface). Minor usability issues (e.g., small buttons) could be overcome using alternative views or intuitive customization capabilities (e.g., zooming in with pinch gestures). Our PD design including graphical visualizations of the individual health data was appropriate for and appreciated by the vast majority of our patients as it allowed them to receive very quickly an overview on their current health status and daily tasks. By using the UEQ, which assesses user experience KPI and has recently also been applied in eHealth evaluations [9, 27], we received valuable responses supporting the definition of precise and transparent goals for further developments of efficient eHealth ICT platforms. Some of these (e.g., better decision support, alerts for physicians when patients show values above resp. below thresholds as defined in their care plan, integration of additional structured patient data, i.e., necessary diagnostic, referral letters) are well-known needs for eHealth applications [28, 29] but are still often missing in existing solutions. We implemented alerts, e.g., in case of duplicate prescriptions, selected medication warnings (i.e., necessary contraception, stopping medication due to scheduled diagnostic procedures), and selected guideline mismatches that are linked to patient’s individual care plan. Similar to an evaluation of an integrated care platform also developed within Horizon2020, patients rated the UX of PICASO overall quite positively and physicians lower [30]. Although patients and physicians experienced performance issues which were due mainly to secure handling of network traffic and GDPR-compliant certificate management that could be improved but not fully solved in project duration, our physicians’ UX experiences might have been more constrained by the evolving system, reflected in lower satisfaction with the platform than among patients. Another quality feature of the platform and the user interface is that it stimulated nearly half of the patients to set new goals for themselves reflecting patients’ empowerment. This is in line with findings from Ragouzeos et al. using a human-centered design to empower rheumatoid arthritis patients through PROMs [31]. They concluded that presenting data graphically on a dashboard seems to be of large value, and communication around PROs and shared goals might be facilitated [31].

Other dashboards and platforms designed to support rheumatologists have been developed [26, 32–35]. Rheum4U offers PROM assessments and is valued for evaluations of treat to target efforts in RA [33], whereas Rheum-PACER integrates and reassembles information from four disparate PRO data sources into actionable views and functions [34]. The eHealth platform (Sanoia®), which enables RA patients’ self-assessment of health and disease status, led to a small improvement in patient-perceived patient-physician interactions [35], as we also observed in our PICASO study. A recent systematic review focused on asynchronous mobile health interventions in RA showing that overall significant beneficial results and desirable outcomes can be postulated [36]. But such software developments, in contrast to PICASO, have so far mostly been implemented only for a specific part of the care continuum. Our PICASO ICT platform integrated data from different care sources, visualized them according to given consent levels, and leave the data at the data owner. To achieve this aim, we involved all relevant target end-users and health care system stakeholders in the development from the very beginning and implemented a usability engineering process. This design approach has been recommended before as it ensures that the platform meets the needs of all stakeholders mentioned above, while allowing IT developers to manage expectations using new IT standards [37–39].

All PICASO services were implemented in conformance with the established HL7/FHIR standard in order to enable modern data exchange with other software systems, a highly needed prerequisite for IT developments in the health care sector [40]. This standard ensured that data from other systems can be orchestrated in the platform and displayed in PDs and CDs. Integration of PICASO into the existing hospital’s (technical) infrastructures and use in patients’ private environments was feasible. The GDPR-conform, secure, and accurate handling of data and data sharing in PICASO is an elementary prerequisite for use in routine patient care [7, 8] as data security is also for patients a very important issue. Our participants rated their confidence in the reliability of Internet information lower than in our last survey [41] and two-thirds of our patients considered the security of data in clouds to be rather low. Nevertheless, these concerns did not deter them from participating in the study and using a novel IT platform with provision of self-reported data.

Overall, the benefits of digital health applications are increasingly adopted to support health care, as they offer opportunities to improve knowledge and create new, optimized management processes [42, 43]. They still have to prove a positive impact on care and outcomes, especially in long-term use [40]. Continuous use of such platforms like PICASO, with regular documentation of comprehensive rheumatologic and cardiovascular measurements, will result in a big data scenario. This facilitates additional developments of AI models to optimize multimorbidity research and management (e.g., RA-specific cardiovascular risk profiles).

### Limitations

Due to the iterative approach and a continuous development process, changes on the ICT platform and its functionalities were prevalent. This limited evaluation and might flatten learning experiences especially by physicians who did not use the system daily like the patients. However, it led to better evaluations towards the end of the project. Obviously, this is a common finding in Horizon2020 projects [30]. In addition, our data represent data from a tertiary center and a small cohort, where one-third of the patients had high education levels, known to increase the willingness to use new technologies [44]. Cautious generalization of the results is required, especially for populations with different characteristics, such as social, economic, or technological marginalization, as, i.e., in developing countries. Therefore, studies in larger and diverse cohorts and different clinical settings over longer periods are warranted. We only tested the platform with selected medical devices handed out from the PICASO team. Hence, different evaluation results with a “bring your own device” approach cannot be excluded. The dashboard approach may need to be adjusted if additional variables not previously considered are to be integrated.

## Conclusions

The PICASO platform offers a modern GDPR-compliant solution that can be implemented in existing infrastructures, leaving data with the owner. The comprehensive aggregation of clinical data from distributed sources improves physicians’ and patients’ knowledge of the rheumatic disease and related comorbidities, as well as their management processes. It preserves and supports existing health resources from both the patients' and physicians' perspective and empowers patients to monitor and positively contribute to their disease management. Further research in larger sample sizes and over longer periods of time is warranted to evaluate the ICT platform effects on patients’ outcome, behavior, and changes in the existing care structures of the health care system.

## Data Availability

The data are available on reasonable request. Project name: PICASO – A Personalized Integrated Care Approach for Service Organisations and Care Models For Patients with Multi-Morbidity and Chronic Condition Project home page: http://picaso-project.eu Funding This project received funding from the European Union’s Horizon 2020 research and innovation program under grant agreement no. 689209. Open Access funding enabled and organized by Projekt DEAL.
